# Supplementary material for: A highly-contiguous genome assembly of the Eurasian spruce bark beetle, Ips typographus, provides insight into a major forest pest
Source: Commun Biol. 2021 Sep 9;4:1059. doi: 10.1038/s42003-021-02602-3 (PMC8429705; doi:10.1038/s42003-021-02602-3)
Supplement: Supplementary file 2 — Supplementary Material [file 42003_2021_2602_MOESM2_ESM.pdf]

A highly-contiguous genome assembly of the Eurasian spruce bark beetle, *Ips typographus*, provides insight into a major forest pest

Daniel Powell, Ewald Groβe-Wilde, Paal Krokene, Amit Roy, Amrita Chakraborty, Christer Löfstedt, Heiko Vogel, Martin N. Andersson and Fredrik Schlyter

Supplementary Information

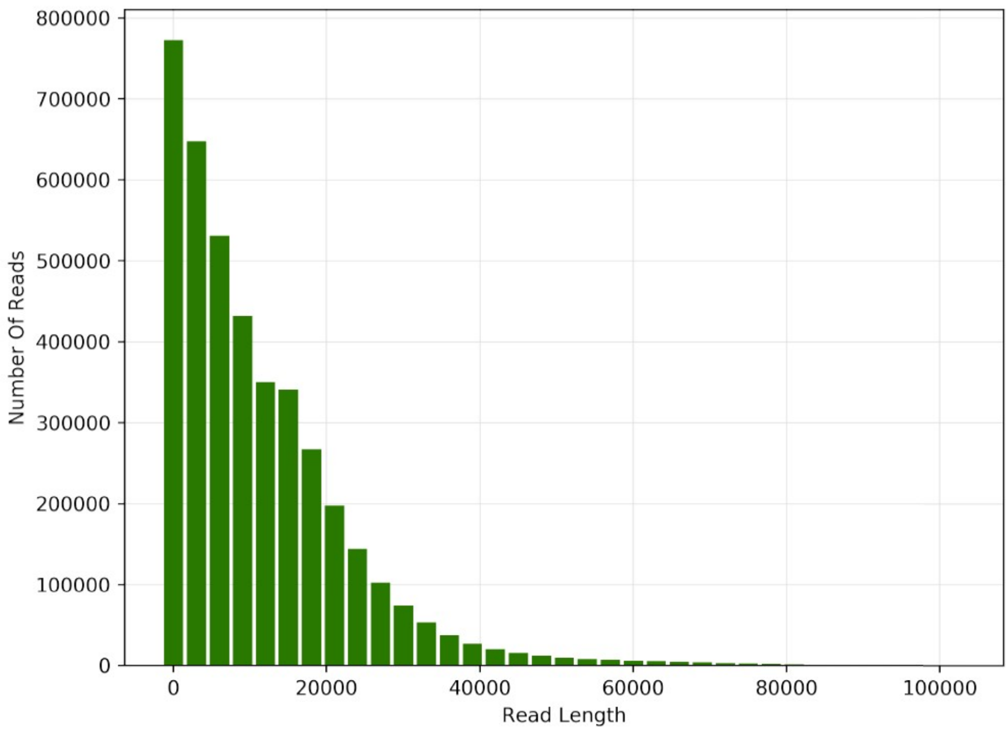

Supplementary Figure 1. Read length distribution from PacBio sequencing of the *Ips typographus* genome.

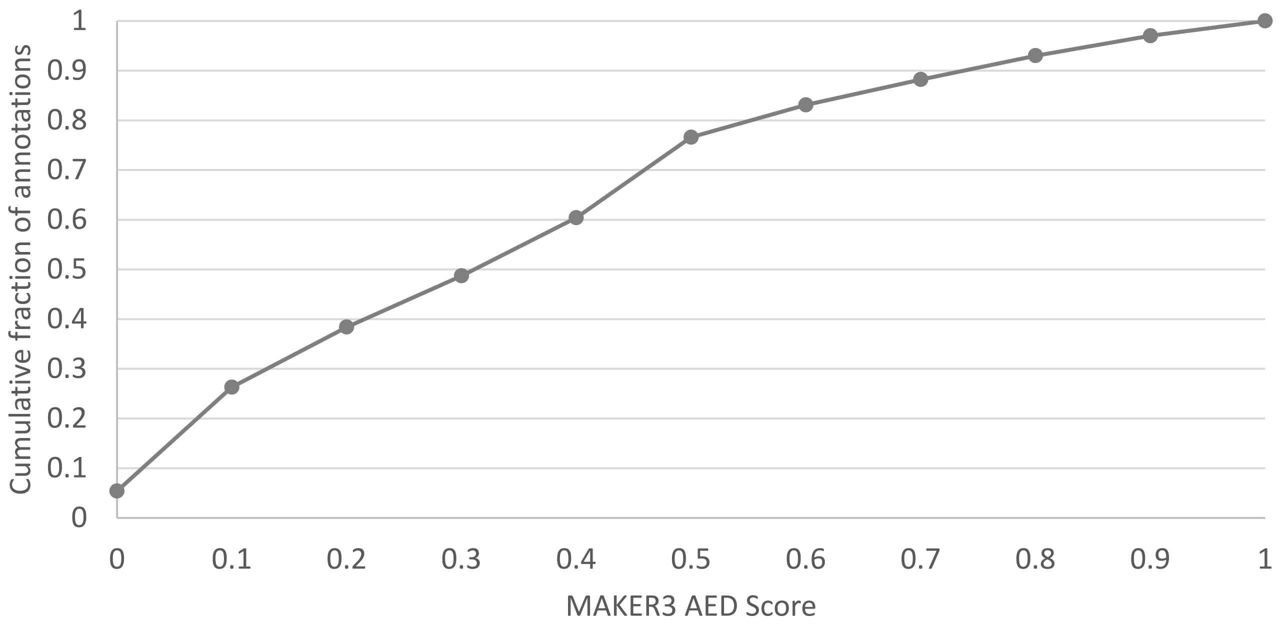

Supplementary Figure 2. Cumulative Annotation Edit Distance (AED) scores for gene models produced using the MAKER3 pipeline. Almost 80% of the gene models have an AED score of 0.5 or less. The AED score is a metric assigned to a gene model by MAKER3 and is a measure of the degree of fit that model has with the supporting evidence.

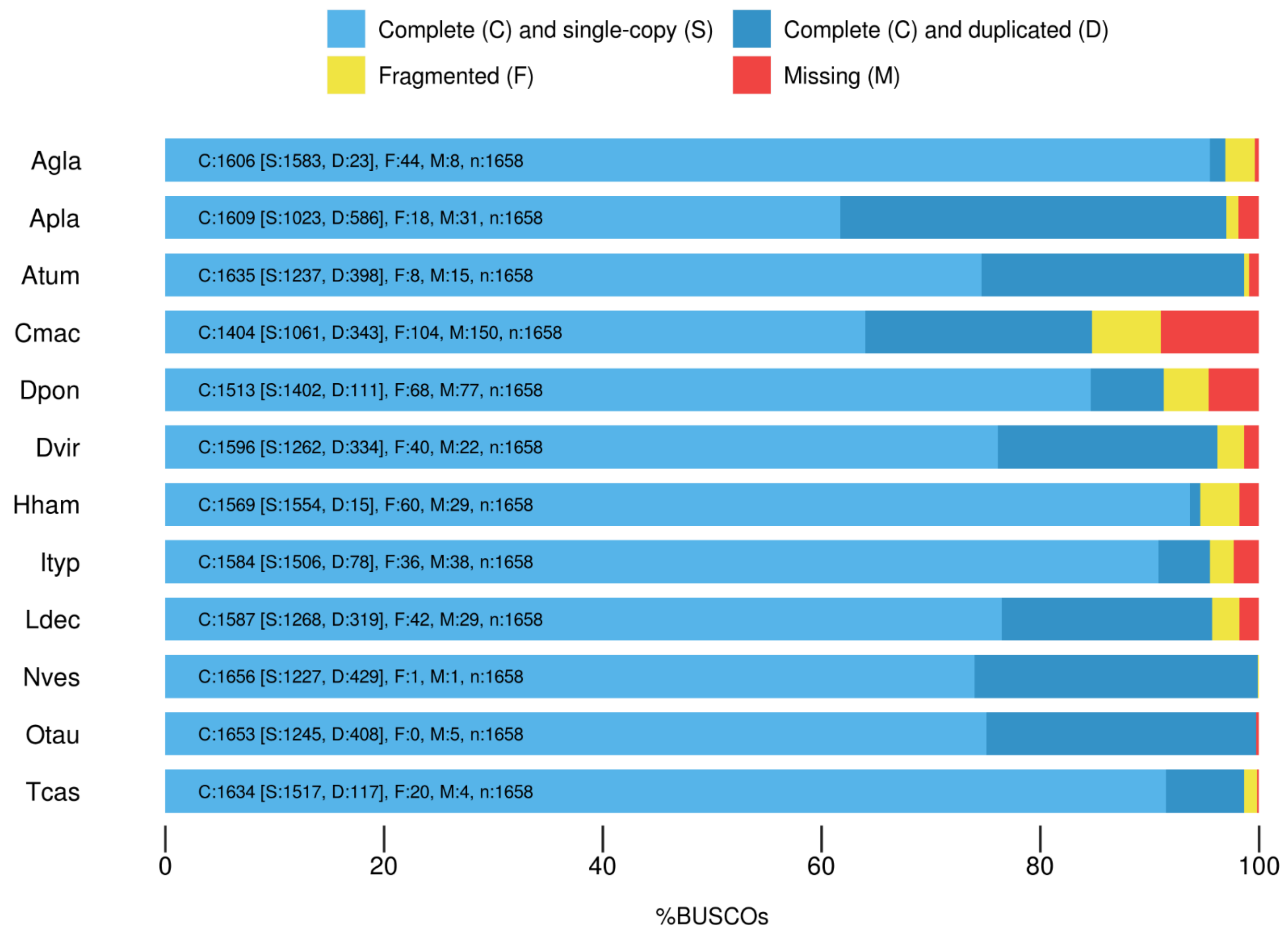

Supplementary Figure 3. Completeness estimates of *Ips typographus* (Ityp) gene models and comparison with other published coleopteran gene sets using BUSCO tool searches against the Insecta dataset of 1,658 genes. Atum, *Aethina tumida*; Apla, *Agrilus planipennis*; Agla, *Anoplophora glabripennis*; Cmac, *Callosobruchus maculatus*; Dpon, *Dendroctonus ponderosae*; Dvir, *Diabrotica virgifera*; Hham, *Hypothenemus hampei*; Ityp, *Ips typographus*; Ldec, *Leptinotarsa decemlineata*; Nves, *Nicrophorus vespilloides*; Otau, *Onthophagus taurus*; Tcas, *Tribolium castaneum.*

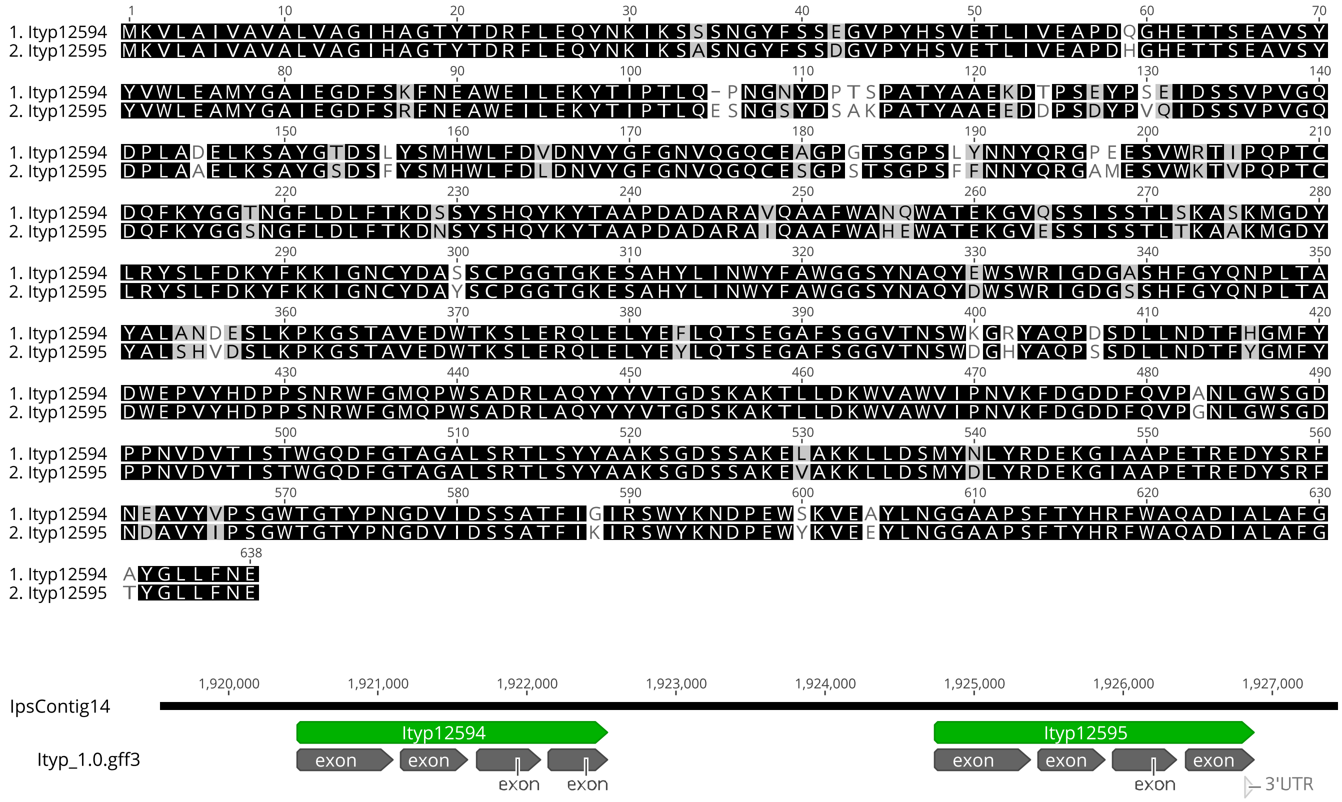

Supplementary Figure 4. Alignment and location of GH48 genes Ityp12594 and Ityp12595 occurring tandemly in the *Ips typographus* genome. Genes are separated by 2,192 bp.

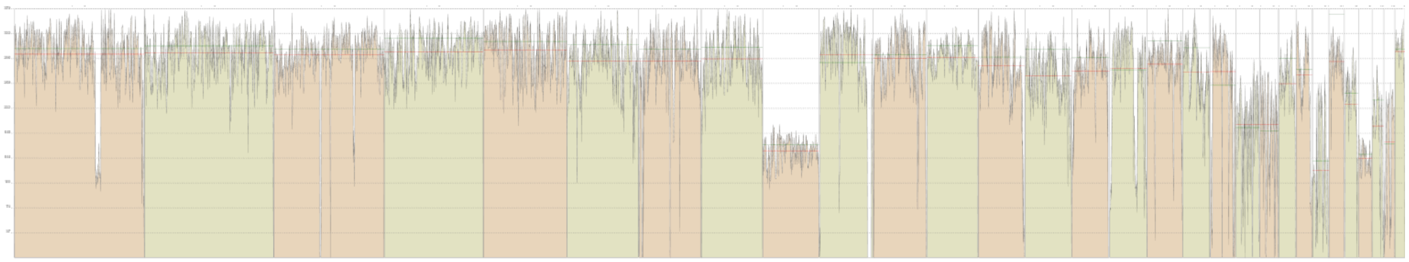

Supplementary Figure 5. Coverage plot of the largest 30 contigs in the *Ips typographus* assembly ordered from largest (left) to smallest (right). Short reads were mapped to the genome assembly from a single male sample. Coverage is relatively even across these largest contigs which contain over 75% of the genome assembly. However, the male being of the heterogametic sex, contigs with approximately half the level of coverage were likely segments of the x chromosome. The *I. typographus* karyotype is 14 + Xy_p. Plot displays the degree of coverage on the y axis and contig on the x axis. Y axis ranges from 0 to 370. Plot was generated with the wgscoverageplotter script (Lindenbaum, Pierre (2015): JVarkit: java-based utilities for Bioinformatics. http://dx.doi.org/10.6084/m9.figshare.1425030)

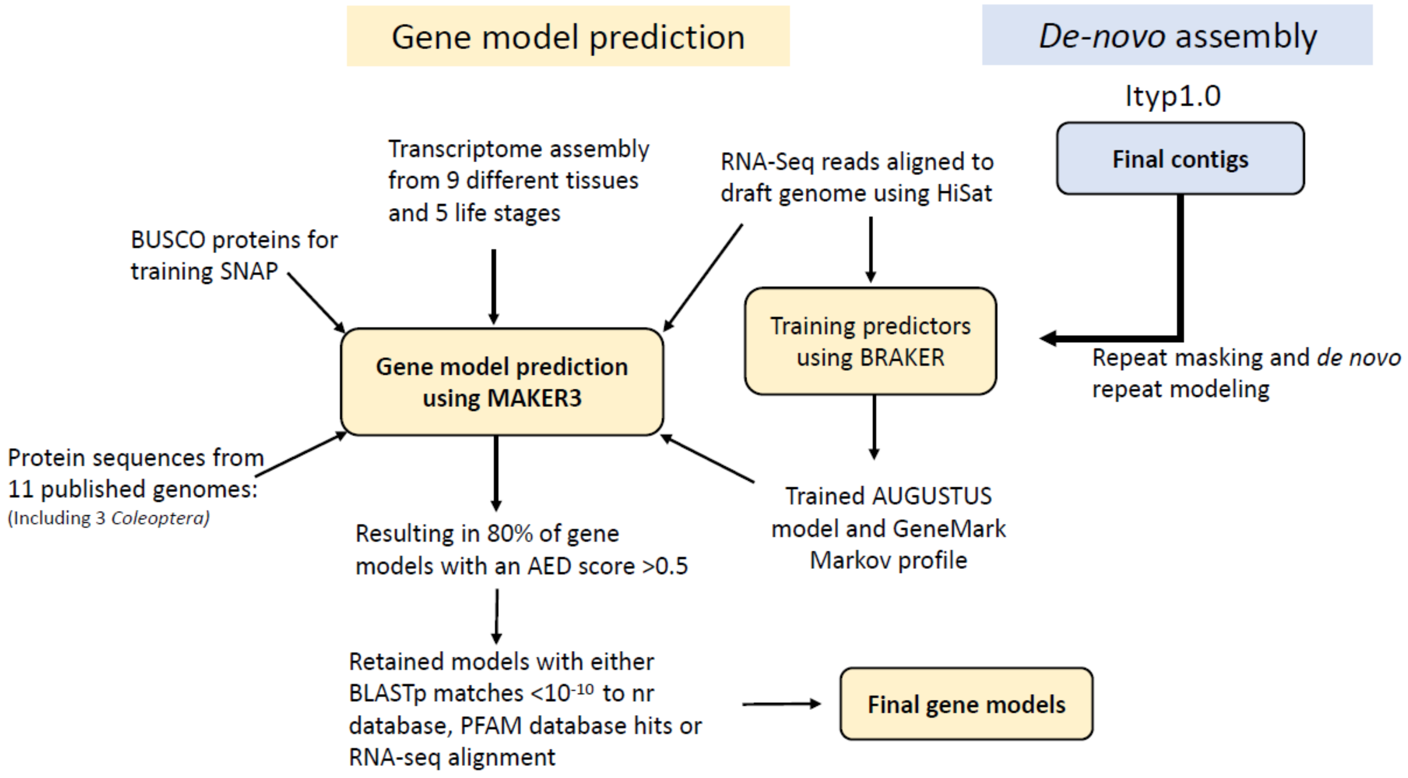

Supplementary Figure 6. Workflow diagram of the gene model prediction pipeline for the *Ips typographus* genome.

Supplementary Table 1. Contig size statistics from PacBio sequencing of the *Ips typographus* genome.

Minimum contig length	Number of contigs	Total contig length
All	272	236,816,287
10 KB	272	236,816,287
25 KB	252	236,352,854
50 KB	190	234,082,415
100 KB	164	232,251,227
250 KB	101	222,160,924
500 KB	72	212,556,464
1 MB	36	186,489,602
2.5 MB	20	161,077,642
5 MB	14	136,853,431
10 MB	5	71,627,877

Supplementary Table 2. Identification of telomeric regions ($TTAGGG_N$) in the *Ips typographus* genome assembly.

Contig ID	Location	Contig Size (Mb)
IpsContig1	Reverse (end of sequence)	16.86
IpsContig2	Forward (start of sequence)	16.77
IpsContig3	Forward (start of sequence)	14.31
IpsContig4	Forward (start of sequence)	12.83
IpsContig5	Reverse (end of sequence)	10.84
IpsContig23	Forward (start of sequence)	2.10
IpsContig51	Forward (start of sequence)	0.76
IpsContig93	Reverse (end of sequence)	0.28

Telomeres found: 8 (5 forward, 3 reverse)

Supplementary Table 3. Classification of annotated repetitive elements identified in the genome of *Ips typographus*.

Repeat class	Number of elements	Total length (bp)*	Percentage (%)*
SINE	52	2806	0.00
LINE	13454	6636866	2.80
LTR elements	20437	8977540	3.79
DNA elements	41770	10228794	4.32
Other	127687	41046601	17.32
Small RNA	521	116873	0.05
Satellites	248	99343	0.04
Simple repeats	31033	1270655	0.54
Low complexity	8129	392333	0.17
**Total bases masked**		**66809513**	**28.20**

*The sum of the repeat class length and percentage values are greater than the total of the repeats masked in the genome due to overlaps between annotated repeats of different classes.

Supplementary Table 4. Extended gene model annotation statistics for the *Ips typographus* genome.

Total sequence length	236,816,287
Number of genes	23,923
Number of exons	121,796
Number of introns	97,873
Number of CDS	23,923
Overlapping genes	5,676
Contained genes	2,154
Total gene length (bp)	132,911,182
Total exon length (bp)	39,477,432
Total intron length (bp)	93,629,496
Total CDS length (bp)	29,963,361
Shortest gene (bp)	135
Shortest exon (bp)	1
Shortest intron (bp)	4
Shortest CDS (bp)	108
Longest gene (bp)	318,767
Longest exon (bp)	85,977
Longest intron (bp)	125,082
Longest CDS (bp)	75,285
Mean gene length (bp)	5,556
Mean exon length (bp)	324
Mean intron length (bp)	957
Mean CDS length (bp)	1,252
Percent of genome covered by genes	56

Supplementary Table 5. Gene ontology enrichment of the 811 orthologous gene clusters unique to *Ips typographus* when compared with *Dendroctonus ponderosae*, *Hypothenemus hampei and Anoplophora glabripennis*.

GO ID	# Clusters	GO Description	GO Category	FDR
GO:0003964	17	RNA-directed DNA polymerase activity	MF	1.07738e-20
GO:0046051	2	UTP metabolic process	BP	0.000447
GO:0004518	5	nuclease activity	MF	0.000461
GO:0045739	3	positive regulation of DNA repair	BP	0.000719
GO:0007166	3	cell surface receptor signalling pathway	BP	0.000874
GO:0006313	6	transposition, DNA-mediated	BP	0.000889
GO:0015074	5	DNA integration	BP	0.001372
GO:0031936	2	negative regulation of chromatin silencing	BP	0.001454
GO:0042803	2	protein homodimerization activity	MF	0.002154

Supplementary Table 6. Summary of the published genomes used in this study.

<u>**Coleoptera > Polyphaga**</u>			***Taxonomy***			***Genome Stats***		
**Species**	**ID**	**# Prot seqs**	**Superfamily**	**Subfamily**	**Common Name**	**Scaffold N50**	**Total Size**	**Genbank Accession**
Aethina tumida	*Atum*	17,463	Cucujoidea	Nitidulinae	Small hive beetle	298,879	234 Mb	GCF_001937115.1
Agrilus planipennis	*Apla*	22,159	Buprestoidea	Agrilinae	Emerald ash borer	1,113,421	353 Mb	GCF_000699045.2
Anoplophora glabripennis	*Agla*	22,343	Chrysomelidae	Lamiinae	Asian longhorned beetle	678,234	706 Mb	GCF_000390285.2
Callosobruchus maculatus	*Cmac*	31,345	Chrysomelidae	Bruchinae	Cowpea weevil	212,245	1.007 Gb	GCA_900659725.1
Dendroctonus ponderosae	*Dpon*	13,457	Curculionoidea	Scolytinae	Mountain pine beetle	628,732	252 Mb	GCF_000355655.1
Diabrotica virgifera virgifera	*Dvir*	28,061	Chrysomeloidea	Galerucinae	Western corn rootworm	489,108	2.418 Gb	GCF_003013835.1
Hypothenemus hampei	*Hham*	19,222	Curculionoidea	Scolytinae	Coffee berry borer	44,715	162 Mb	GCA_013372445.1
Ips typographus	***Ityp***	**23,937**	**Curculionoidea**	**Scolytinae**	**Eurasian spruce bark beetle**	**6,654,004**	**236 Mb**	**This study**
Leptinotarsa decemlineata	*Ldec*	19,038	Chrysomeloidea	Chrysomelinae	Colorado potato beetle	139,046	641 Mb	GCF_000500325.1
Nicrophorus vespilloides	*Nves*	19,577	Staphylinoidea	Nicrophorinae	Burying beetle	122,407	195 Mb	GCF_001412225.1
Onthophagus taurus	*Otau*	21,668	Scarabaeoidea	Scarabaeinae	Taurus scarab	337,157	267 Mb	GCF_000648695.1
Tribolium castaneum	*Tcas*	18,534	Tenebrionoidea		Red flour beetle	4,456,720	165 Mb	GCF_000002335.3

Supplementary Table 7. Full names of protein families and corresponding PFam identifiers.

**PF00295**	Glyco_hydro_28	Glycosyl hydrolases family 28
**PF00413**	Peptidase_M10	Matrixin
**PF01095**	Pectinesterase	Pectinesterase
**PF02011**	Glyco_hydro_48	Glycosyl hydrolase family 48
**PF02015**	Glyco_hydro_45	Glycosyl hydrolase family 45
**PF10545**	MADF_DNA_bdg	Alcohol dehydrogenase transcription factor Myb/SANT-like
**PF13837**	Myb_DNA-bind_4	Myb/SANT-like DNA-binding domain
**PF14683**	CBM-like	Polysaccharide lyase family 4, domain III
**PF05485**	THAP	THAP domain
**PF00098**	zf-CCHC	Zinc knuckle
**PF01471**	PG_binding_1	Putative peptidoglycan binding domain
**PF05869**	Dam	DNA N-6-adenine-methyltransferase (Dam)
**PF09337**	zf-H2C2	H2C2 zinc finger
**PF13650**	Asp_protease_2	Aspartyl protease
**PF13873**	Myb_DNA-bind_5	Myb/SANT-like DNA-binding domain
**PF14492**	EFG_II	Elongation Factor G, domain II
**PF14686**	fn3_3	Polysaccharide lyase family 4, domain II

Supplementary Table 8. Details of RNA-Seq samples used in this study.

Sample ID	Description	Number of PE reads	% Mapping to genome
L1	larvae stage 1	171,286,469	95.76%
L2	larvae stage 2	159,867,283	94.62%
L3	larvae stage 3	159,565,350	93.79%
P	pupae	160,837,226	93.98%
AFM	adult beetle (male & female)	181,374,351	93.59%
AMFG	fed adult male gut	187,490,572	93.25%
AMFH	fed adult male head	173,389,571	92.18%
CFFB	callow female beetle fat body	245,181,256	93.95%
CFG	callow female beetle gut	189,231,418	95.26%
CFH	callow female beetle head	159,092,068	89.57%
CMFB	callow male beetle fat body	184,072,277	93.12%
CMG	callow male beetle gut	174,127,755	94.95%
CMH	callow male beetle head	188,654,217	92.25%
