# Supplementary material for: A highly-contiguous genome assembly of the Eurasian spruce bark beetle, Ips typographus, provides insight into a major forest pest
Source: Commun Biol. 2021 Sep 9;4:1059. doi: 10.1038/s42003-021-02602-3 (PMC8429705; doi:10.1038/s42003-021-02602-3)
Supplement: Supplementary file 3 — Reporting summary [file 42003_2021_2602_MOESM3_ESM.pdf]

nature research

Corresponding author(s): Martin N. Andersson

Last updated by author(s): Aug 7, 2021

Reporting Summary

Nature Research wishes to improve the reproducibility of the work that we publish. This form provides structure for consistency and transparency in reporting. For further information on Nature Research policies, see our Editorial Policies and the Editorial Policy Checklist.

Statistics

For all statistical analyses, confirm that the following items are present in the figure legend, table legend, main text, or Methods section.

n/a	Confirmed	
☐	☒	The exact sample size (*n*) for each experimental group/condition, given as a discrete number and unit of measurement
☒	☐	A statement on whether measurements were taken from distinct samples or whether the same sample was measured repeatedly
☐	☒	The statistical test(s) used AND whether they are one- or two-sided<br>*Only common tests should be described solely by name; describe more complex techniques in the Methods section.*
☒	☐	A description of all covariates tested
☐	☒	A description of any assumptions or corrections, such as tests of normality and adjustment for multiple comparisons
☒	☐	A full description of the statistical parameters including central tendency (e.g. means) or other basic estimates (e.g. regression coefficient) AND variation (e.g. standard deviation) or associated estimates of uncertainty (e.g. confidence intervals)
☐	☒	For null hypothesis testing, the test statistic (e.g. *F*, *t*, *r*) with confidence intervals, effect sizes, degrees of freedom and *P* value noted<br>*Give P values as exact values whenever suitable.*
☒	☐	For Bayesian analysis, information on the choice of priors and Markov chain Monte Carlo settings
☒	☐	For hierarchical and complex designs, identification of the appropriate level for tests and full reporting of outcomes
☐	☒	Estimates of effect sizes (e.g. Cohen's *d*, Pearson's *r*), indicating how they were calculated

Our web collection on statistics for biologists contains articles on many of the points above.

Software and code

Policy information about availability of computer code

Data collection	Raw sequence data was processed using either Illumina or PacBio systems software relevant for each platform.
Data analysis	The publicly availably software used in this study are: FALCON-kit v1.3.0; FALCON-unzip v1.2.0; MUMmer; BUSCO v3.0.2; FindTelomeres.py; Jellyfish v2.3.0; GenomeScope 2.0; RepeatModeler v1.0.11; RepeatMasker v4.0.8; HiSat2 v2.1.0; StringTie v1.3.3; BRAKER2; GeneMark-ET; MAKER3; SNAP; AUGUSTUS; BLAST+; HMMER v3.1; OrthoVenn2; OrthoFinder v2.4.0; FigTree v1.4.4; Trimmomatic v0.36; Trinity v2.8.2; SAMtools v1.5; CAFE v4.2.1 R. Details of usage including parameters are described in the materials and methods. Direct links are available for all software that are not able to be cited.

For manuscripts utilizing custom algorithms or software that are central to the research but not yet described in published literature, software must be made available to editors and reviewers. We strongly encourage code deposition in a community repository (e.g. GitHub). See the Nature Research guidelines for submitting code & software for further information.

Data

Policy information about availability of data

All manuscripts must include a data availability statement. This statement should provide the following information, where applicable:

- Accession codes, unique identifiers, or web links for publicly available datasets
- A list of figures that have associated raw data
- A description of any restrictions on data availability

This Whole Genome Shotgun project has been deposited at DDBJ/ENA/GenBank under the accession JADDUH000000000. The version described in this paper is version JADDUH010000000. All sequence data relating to this study are available under the BioProject accession numbers PRJNA671615 and PRJNA679450. Source data used to construct the figures in this manuscript have been uploaded to the Figshare repository: https://doi.org/10.6084/m9.figshare.14503065

Field-specific reporting

Please select the one below that is the best fit for your research. If you are not sure, read the appropriate sections before making your selection.

☐ Life sciences ☐ Behavioural & social sciences ☒ Ecological, evolutionary & environmental sciences

For a reference copy of the document with all sections, see nature.com/documents/nr-reporting-summary-flat.pdf

Ecological, evolutionary & environmental sciences study design

All studies must disclose on these points even when the disclosure is negative.

Study description	This paper describes the genome assembly, annotation and comparative analysis of the Eurasian spruce bark beetle, Ips typographus, with 11 other species of coleoptera. We identify gene family expansions associated with plant cell wall degradation and map the expression of these genes across tissues and life stages. We also describe an approach for generating larger quantities of HMW DNA from pooled samples from an inbred population.
Research sample	Laboratory-reared population of I. typographus kept at the Swedish University of Agricultural Sciences in Alnarp, Sweden, originating from individuals collected 1983 in Lardal, Norway. Additionally, wild beetles were collected from naturally infested logs field, Czech Republic, for use in RNA-Seq experiments.
Sampling strategy	DNA was extracted from around 100 adult males from this 10× inbred population and used for library preparation. RNA was extracted from different life stages and tissues.
Data collection	Data was generated at the sequencing facility at the Uppsala Genome Center, Science for Life Laboratory, Uppsala University, Sweden
Timing and spatial scale	DNA samples were obtained at a single time point from the same generation of a laboratory population.
Data exclusions	Illumina data generated for a genome survey were not included in the assembly or for downstream analysis and only used for genome size estimation using kmer counts.
Reproducibility	PacBio shotgun WGS was performed once on the total pool of beetle DNA. Gene annotation pipelines were iteratively processed with progress assessed after each run.
Randomization	Randomization was not relevant to this study.
Blinding	Blinding was not relevant to this study.

Did the study involve field work? ☐ Yes ☒ No

Reporting for specific materials, systems and methods

We require information from authors about some types of materials, experimental systems and methods used in many studies. Here, indicate whether each material, system or method listed is relevant to your study. If you are not sure if a list item applies to your research, read the appropriate section before selecting a response.

Materials & experimental systems

n/a	Involved in the study	
☒	☐	Antibodies
☒	☐	Eukaryotic cell lines
☒	☐	Palaeontology and archaeology
☐	☒	Animals and other organisms
☒	☐	Human research participants
☒	☐	Clinical data
☒	☐	Dual use research of concern

Methods

n/a	Involved in the study	
☒	☐	ChIP-seq
☒	☐	Flow cytometry
☒	☐	MRI-based neuroimaging

Animals and other organisms

Policy information about studies involving animals; ARRIVE guidelines recommended for reporting animal research

Laboratory animals	A continuous culture of I. typographus was kept at the Swedish University of Agricultural Sciences in Alnarp, Sweden originating 1983 from wild caught animals from Lardal, SW Norway.
Wild animals	Wild beetles were obtained from naturally infested logs field, Czech Republic.

Field-collected samples	RNA from F1 adults emerging in the lab from naturally infested logs from the field, Czech Republic.
Ethics oversight	No animal ethics was required for this study involving only arthropods.

Note that full information on the approval of the study protocol must also be provided in the manuscript.
